# Automatic Classification of Magnetic Resonance Histology of Peripheral Arterial Chronic Total Occlusions Using a Variational Autoencoder: A Feasibility Study

**DOI:** 10.3390/diagnostics13111925

**Published:** 2023-05-31

**Authors:** Judit Csore, Christof Karmonik, Kayla Wilhoit, Lily Buckner, Trisha L. Roy

**Affiliations:** 1 DeBakey Heart and Vascular Center, Houston Methodist Hospital, 6565 Fannin Street, Houston, TX 77030, USA; 2 Heart and Vascular Center, Semmelweis University, 68 Városmajor Street, 1122 Budapest, Hungary; 3 MRI Core, Translational Imaging Center, Houston Methodist Research Institute, 6670 Bertner Avenue, Houston, 77030 TX, USA; ckarmonik@houstonmethodist.org (C.K.);

**Keywords:** peripheral arterial disease, ultra-high field magnetic resonance imaging, endovascular treatment, non-invasive diagnostics, chronic limb-threatening ischemia, angioplasty, angiography, chronic total occlusion

## Abstract

The novel approach of our study consists in adapting and in evaluating a custom-made variational autoencoder (VAE) using two-dimensional (2D) convolutional neural networks (CNNs) on magnetic resonance imaging (MRI) images for differentiate soft vs. hard plaque components in peripheral arterial disease (PAD). Five amputated lower extremities were imaged at a clinical ultra-high field 7 Tesla MRI. Ultrashort echo time (UTE), T1-weighted (T1w) and T2-weighted (T2w) datasets were acquired. Multiplanar reconstruction (MPR) images were obtained from one lesion per limb. Images were aligned to each other and pseudo-color red-green-blue images were created. Four areas in latent space were defined corresponding to the sorted images reconstructed by the VAE. Images were classified from their position in latent space and scored using tissue score (TS) as following: (1) lumen patent, TS:0; (2) partially patent, TS:1; (3) mostly occluded with soft tissue, TS:3; (4) mostly occluded with hard tissue, TS:5. Average and relative percentage of TS was calculated per lesion defined as the sum of the tissue score for each image divided by the total number of images. In total, 2390 MPR reconstructed images were included in the analysis. Relative percentage of average tissue score varied from only patent (lesion #1) to presence of all four classes. Lesions #2, #3 and #5 were classified to contain tissues except mostly occluded with hard tissue while lesion #4 contained all (ranges (I): 0.2–100%, (II): 46.3–75.9%, (III): 18–33.5%, (IV): 20%). Training the VAE was successful as images with soft/hard tissues in PAD lesions were satisfactory separated in latent space. Using VAE may assist in rapid classification of MRI histology images acquired in a clinical setup for facilitating endovascular procedures.

## 1. Introduction

Due to its minimally invasive nature and low periprocedural complication rate, percutaneous vascular intervention (PVI) is frequently used as a first-line therapy for peripheral arterial disease (PAD). However, immediate PVI failure, however, occurs in 20% of patients due to impenetrable plaques [1]. Moreover, individuals who require bypass surgery after first PVI failure have greater amputation rates and lower long-term patency than those who initially received bypass surgery [2].

The management of peripheral artery disease is limited by current imaging modalities [3]. One of the research priorities laid out in the Global Vascular Guidelines of the Society for Vascular Surgery (SVS) for chronic limb-threatening ischemia (CLTI) is the need for improvement of non-invasive imaging using magnetic resonance imaging (MRI) [4]. Poor calcium sensitivity of MRI has been a major barrier to the clinical adoption of the technique for chronic total occlusion (CTO) applications. Recent advances in ultrashort echo time (UTE) MRI have been used to separate calcium from other tissues which contain protons but possess a short T2* [5,6,7,8,9,10]. In particular for peripheral atherosclerotic lesions, UTE was shown to distinguish between soft tissue types (including fat, thrombus, microchannels, or loose fibrous tissue) as hyperintense and hard tissue types (dense collagen and/or speckled calcium signals and calcified nodules) as isointense and calcium as hypointense to relative to smooth muscle tissue signal [11]. Conventional imaging contrasts such as T1-weighted (T1w) or T2-weighted (T2w) will yield little to no signal for hard tissue types due to short T2* value and no signal for calcium.

The goal of this study was to develop an automated classification procedure for the images acquired of a PAD lesion with a dedicated high-resolution MRI histology protocol that utilizes UTE, T1w, and T2w contrasts. Artificial intelligence (AI) algorithms based on neural networks are able to classify complex data interrelationships in general [12,13,14,15,16,17,18,19,20,21,22]. Certain AI algorithms convert information in medical image data to features by a dense representation and so enable classifications with unsupervised learning. One of these are variational autoencoders (VAEs) [23,24,25,26,27,28,29] which create a latent space with a reduced dimension that has also been applied to medical imaging data [30,31,32,33,34,35,36,37,38,39,40,41,42,43]. The latent space can then serve to perform semi-supervised classification of the complex information contained in the input data [44,45,46,47,48,49]. For two-dimensional image data, a two-dimensional (2D) convolutional neural network (CNN) integrated into the VAE creates the latent space. The latent space serves as input for a reversed 2D CNN to recreate the original images. The representation of the condensed data in the latent space can then serve as a means for the classification of the original images. In a novel approach, our study is investigating the feasibility of the application of the VAE algorithm on multi-contrast MRI images acquired with a specialized MRI-histology protocol. CNN layers were customized and adapted to the goal of achieving a sufficient separation of the reconstructed images containing soft and hard tissues in latent space.

To train the VAE algorithm effectively, a significant amount of data is required. In the case of an MRI dataset, a sufficient number of input images can be obtained through interpolated multiplanar reconstruction (MPR) of a single lesion. The 2D image data can then be transformed into a pseudo-color image, where the grayscale values of the three MRI contrasts (red, green, and blue) are used to represent the color components. These pseudo-color images encompass the two classes of interest, namely hard and soft tissue components. In MRI images, the hard tissue components typically exhibit little to no signal, resulting in dark or black image intensities. Conversely, the soft tissue components generate bright-colored regions due to their signal characteristics. By performing a semi-supervised classification in the latent space, each lesion composed of the pseudo-color images can be assigned a score indicating whether it is predominantly composed of hard or soft tissue. Its presence or absence has previously been related to the guidewire puncture force required to cross the lesion [11] which may aid in the planning of interventional procedures.

In this study, we present a proof-of-concept implementation of the method described above. The approach utilizes high-resolution images obtained through a multi-contrast MRI-histology protocol and employs a 2D CNN-based VAE. The primary objective of this study is to assess the feasibility of the proposed method. We evaluated the ability of the implemented AI algorithm to reconstruct the original images accurately using the condensed information in the latent space. Also, we assessed the effectiveness of semi-supervised classification in distinguishing the presence and absence of hard and soft tissue components. In the future, AI-aided analysis may help rapid, accurate evaluation of lesion crossability in planning endovascular interventions.

## 2. Materials and Methods

This single-center, retrospective study was approved by the Institutional Review Board. Indication of amputation was end-stage chronic limb ischemia and was independent of this current study. All patients provided informed consent. All procedures were carried out in accordance with the Declaration of Helsinki.

### 2.1. Ultra-High Field Magnetic Resonance Imaging of Amputated Limbs

Amputated limbs were harvested immediately after surgery from five patients. Legs were set up in the MRI scanner mimicking a clinical setup. All images were acquired at a Food and Drug Administration (FDA) approved clinical 7T MRI scanner (Siemens Healthineers, Erlangen, Germany) with a single-transmit 28-channel knee coil. The duration of the MRI examination was less than one hour which may be considered tolerable in a clinical setting. The MRI protocol included UTE, T1w and T2w sequences acquired sagittally with the following parameters. UTE (pointwise-encoding time reduction with radial acquisition (PETRA)): field of view (FOV): 150 mm, phase FOV 100%, in-plane resolution 0.2 × 0.2 mm, slice thickness: 0.2 mm, repetition time (TR) 10 ms, echo time (TE) 0.07 ms, flip angle (FA) 4. T2-weighted (Double Echo Steady State (DESS): FOV 160 mm, phase FOV 87.5%, slice thickness 0.2 mm, TR 12.57 ms, TE 6 ms, FA 25, water excitation, 512 slices). T1-weighted (: three-dimensional (3D) Fast Low-Angle Shot (FLASH): FOV 160 mm, phase FOV 87.6%, in-plane resolution 0.2 × 0.2 mm, slice thickness 0.2 mm, TR 9.5 ms, TE 4.09 ms, FA 7, Generalized Auto-Calibrating Partially Parallel Acquisitions (GRAPPA) 2, phase encode direction A >> P, 512 slices, water excitation).

### 2.2. Image Preprocessing

Image volumes were co-registered with the 3D Slicer software [50] using the General Registration module (with the options ‘useMomentsAlign’ and ‘Rigid + Scale, 7 degrees of freedom’). The aligned volumes were exported in Neuroimaging Informatics Technology Initiative (NIFTI) format and loaded into ImageJ (Rasband, W.S., U. S. National Institutes of Health, Bethesda, MD, USA) [51]. Images were normalized across all lesions to equalize image contrasts. Normalized images for the three contrasts were used to create red-green-blue (RGB) images with the T1w images as the red component, the T2w images as the green component and the UTE images as the blue component. Each PAD lesion was manually outlined in the acquired sagittal orientation. The outlined volumes were resliced with the original resolution to create the axial MPR reconstructions. Each axial slice was cropped with the ‘Auto-Crop’ function available in ImageJ and resized to 64 × 64 pixels. For extracting hard/soft (i.e., dark blue/black and blue, respectively) components, pixels with hexadecimal RGB values ranging from 0x800000 to 0xff0000 were removed.

### 2.3. Variational Autoencoder

**Figure 1:**
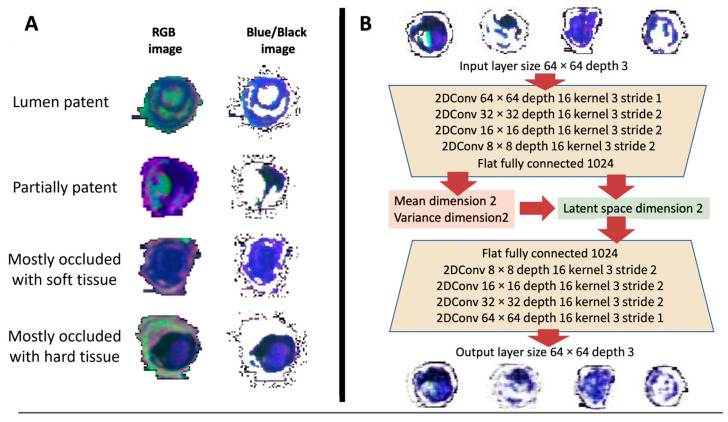
(**A**) Representative cross-sections of pseudo-color Red-Green-Blue (RGB) images are displayed. The RGB images consist of T1-weighted (red), T2-weighted (green), and Ultrashort Echo Time (blue) contrasts. Images correspond to the four different tissue classes located in the latent space of the Two-Dimensional Convolutional Neural Network Variational Autoencoder (2D CNN VAE). (**B**) Schematic illustration of the structure of the 2D CNN VAE, providing the details of the parameters both for the encoder and decoder layers. At the top, representative input cross-sections are shown, while at the bottom the corresponding cross-sections reconstructed by the VAE are displayed. In this basic scheme of a VAE, the model receives the pseudo-color images as input. The encoder component employs multiple layers with varying resolutions, kernel sizes, and strides to compress the input into the latent space. The encoder progressively reduces the dimensionality of the data while extracting relevant features. The decoder receives the information sampled from the latent space as an input. Its purpose is to reconstruct output cross-sections that closely resemble the input performing a series of steps, similar to the encoder, but in the reverse order. 2DConv: Two-Dimensional Convolutional Layer; 2DConvTrans: Two-Dimensional Transposed Convolutional Layer.

Two 2D CNNs were designed with the pre-processed images as input for the first network. From a 2D input layer of depth three for the pseudo-color RGB images, five convolutional layers of increasing depth were created (Figure 1B). The last layer, the output layer, was flat fully connected with the same number of elements as the last convolutional layer. This layer fed into two separate layers, the mean and variance layer, which are of the same dimension as the latent space (two) and represent the mean and variance for two Gaussian distributions. From those, the representation of each image is calculated by a point (x and y component) of that image in latent space. The VAE was implemented on a MacBook Pro with the M1 chip with tensorflow (version 2.7.0) using Python (version 3.9.9) with a virtual environment created with Conda (version 4.11.0). The tensorflow-metal plugin was implemented to allow the use of the Apple M1 Graphics Processing Unit (GPU) approximately accelerate computations by an order of magnitude. With a total of 500 epochs, batch size of 128 and a learning rate of 0.0005 (Adam optimizer) the VAE was trained.

### 2.4. Latent Space Classification and Tissue Scores

From the display of the reconstructed axial segmented hard/soft (black/blue) luminal cross sections in latent space, this space was segmented by inspection into four regions representing four PAD tissue classes, and a numerical tissue score was assigned to each class as follows. Tissue Class 1: lumen patent, tissue score 0; Tissue Class 2: lumen partially patent, tissue score 1; Tissue Class 3: lumen mostly occluded soft tissue, tissue score 3 and Tissue Class 4: lumen mostly occluded hard tissue, tissue score 5 (Figure 1A). An average tissue score was calculated for each lesion sample by averaging the tissue scores for each cross-section and the relative class percentages were determined. In equation form, average tissue score = (sum of individual tissue scores for each axial MPR image of that lesion)/number of MPR images per lesion.

## 3. Results

### 3.1. Image Preprocessing

In total, 2390 MPR reconstructed images from the original five lesion samples (#1: 168 image, #2: 453, #3: 943, #4: 514, #5: 312) were obtained. Pseudo-colors created from the MRI contrasts as described in Section 2.2 ‘Image Preprocessing’ corresponded to arterial wall (red/pink), free lumen/blood (green), calcium (black), collagen (dark blue) and soft tissue (bright blue) (illustrated in Figure 1A). Removal of the RGB pixels as described in Section 2.2 ‘Image Preprocessing’ resulted in remaining black/dark blue and bright blue pixels corresponding to hard and soft tissues (representative images are shown in Figure 1B)

### 3.2. Variational Autoencoder

**Figure 2:**
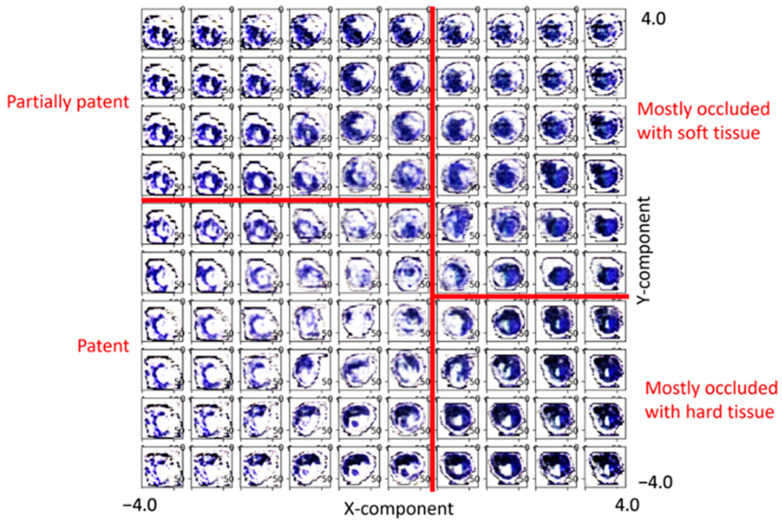
Visualization of distribution of reconstructed cross sections in latent space spawned by x and y-components. Boundaries chosen to separate the latent space into the subregions for the four peripheral artery disease (PAD) lesion Tissue Classes are shown in red. Soft PAD lesion components are presented with blue, hard lesion are presented with black color.

After visual inspection (representative comparison of selected images is presented in Figure 1B), very good agreement between the original and the AI-reconstructed pseudo-color images indicated a good performance of the implementation of the trained 2D CNN VAE which was also found by the good separation of image features (hypointense regions) in the 2D latent space from the reconstructed images based on their spatial location (x coordinates ranging from −4.0 to 4.0, y-coordinates ranging from −4.0 to 4.0 are shown in Figure 2). Colors and their spatial distribution were in good agreement with only light blurring of the reconstructed axial slices present (Figure 1B). From the distribution of the reconstructed axial slices in latent space, a separation of this space into four rectangular regions corresponding to the four tissue classes was defined by assigning boundaries of these regions with values for the x and y-components in latent space as follows: Class 1: lumen patent, x-value < 0.8, y-value < 0.8; Class 2: lumen partially patent; x-value < 0.8, y-value > 0.8; Class 3: lumen mostly occluded soft tissue, x-value > 0.8, y-value ≤ 0.8 and Class 4: lumen mostly occluded hard tissue, x-value > 0.8 and y-value ≤ 0.8 (Figure 2).

### 3.3. Tissue Scores

**Figure 3:**
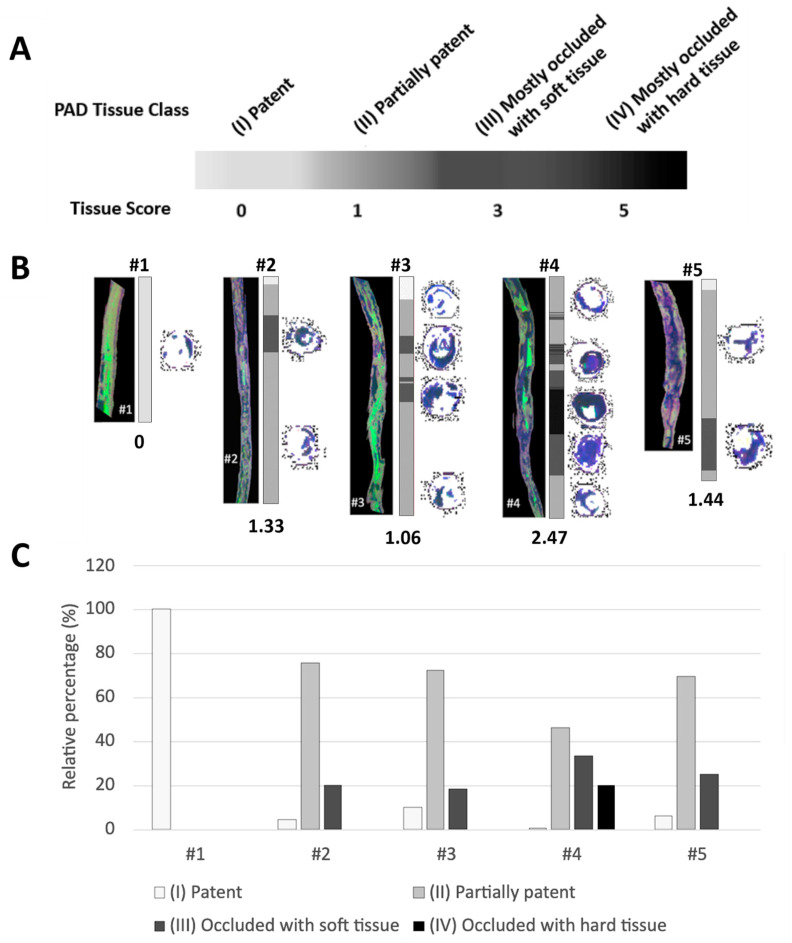
(**A**) Greyscale-coded display of determined Tissue Class based on tissue score for each slice, used for (**B**,**C**). (**B**) Centerline pseudo-color red-green-blue (RGB; T1-weighted—red, T2-weighted—green, Ultrashort Echo Time—blue) images are shown for each of the five lesions (numbered #1–#5) on left. On right, greyscale-coded display of determined Tissue Class for each slice. Average values for the tissue score for the entire lesion are given below. (**C**) Inset displays percentage distribution of tissue scores for each lesion (#1–#5) in same greyscale color. PAD: Peripheral Artery Disease.

The distribution of the tissue scores for the axial slices corresponded well with visual inspection of the distribution of plaque components (from Section 3.1 ‘Image Preprocessing’) for each tissue visible in sagittal cross sections. (displayed in Figure 3). Tissue scores varied in range with tissue sample #1 having an average tissue score value of 0 corresponding to a patent lumen. Lesion sample #3 mostly contained regions that were partially patent (Class 2) with two focal regions with a mostly included lumen (Class 3). Lesion samples #2 and #5 were similar with larger regions corresponding to Class 3 than Class 2. Lesion sample #3 included a large portion of Class 4 surrounded by regions proximal and distal of Class 3 and therefore had the highest tissue score (Figure 3B). Relative percentages of the tissue classes for lesion samples #2-#5 were highest for Class 1 followed by Class 2 and Class 3. For lesion sample 1, only class 1 was present (Table 1 and Figure 3C).

## 4. Discussion

In our study, we investigated the feasibility of applying an AI-motivated semi-automated algorithm for tissue classification on a clinically feasible MRI-histology protocol, which was established for the visualization and the quantification of PAD lesion components. In particular, the presence and extent of hard tissue will present technical challenges for current endovascular treatment options [11] relative to soft tissue components. Standard clinical vascular imaging methods, such as computed tomographic angiography (CTA) or ultrasound, are currently limited in visualizing tissue components of PAD lesions mostly due to limitations in soft tissue contrast beyond the presence of calcium and the presence of stenosis [52,53,54]. MRI is unique in its capability of adjusting soft tissue contrast for atherosclerotic lesions and therefore the ideal methodology for this task [3,11,55,56,57,58]. Commonly used AI algorithms such as VAEs possess the advantage of quick and automated classification, once a particular algorithm has been properly trained. A large amount of data in the range of thousands of images is beneficial for achieving a well-trained algorithm. AI-aided analysis of multimodality carotid and coronary artery plaque tissue characterization and classification has already proven to be an accurate and robust path for facilitating clinical decision-making [59,60], but we only have limited data on using AI for PAD lesion characterization [61]. In our feasibility study, we developed an approach of obtaining several hundred images from one PAD lesion as described. Several kinds of PAD lesions exist. Simple lesions may contain only a small variety of tissue components. With increasing complexity, tissue complexity increases. In severe cases, such as CTI, lesion composition is complex over a large spatial extent which is accessible by high-resolution MRI imaging. Consecutive slices perpendicular to the lesion centerline acquired at a high spatial resolution will contain varying tissue compositions of soft or hard tissues making hundreds of images available per lesion for inclusion into the analysis algorithm. Therefore, a relatively small number of lesions (five) was sufficient to obtain suitable image reconstructions and a good separation of the classes in question (presence and amount of soft and hard tissue components) as evaluated by medical imaging experts and clinical professionals. Limiting the information in the input to the VAE (here the extent of the color space) a-priori reduces the amount of training data for successful semi-supervised classification with the AI-algorithm as well as its design complexity. Layered 2D convolutional layers to create the latent space represent a standard 2D CNN. The parameters for each layer, such as the kernel and stride sizes, as well as the depths of the layers, were optimized for best reconstruction of the original pseudo-color RGB images. If the boundaries for clusters in the latent space are non-linear, it can be challenging to effectively separate the clusters using a two-dimensional latent space. In such cases, using a higher-dimensional latent space may be more feasible and beneficial. In our dataset, images contained two classes of interest which are intended to be distinguished (presence of soft and/or hard tissue). With these limited number of classes, the use of the VAE algorithm and a 2D latent space was found sufficient for the intended image classification, which is apparent by the well-separated reconstructed images by the trained VAE. While a 2D latent space may limit the representation capacity compared to higher-dimensional latent spaces, we wanted to prove that it can still capture these essential features and variations in the data. A higher-dimensional latent space may lead to increased complexity, require more training data and increase the risk of overfitting. It is crucial to strike a balance between the dimensionality of the latent space, model capacity, and the available data resources. However, it is also worth noting that the dimensionality of the latent space is not fixed, and it can be increased if necessary.

Also, using a decoder arm helps in capturing the essential details and characteristics of the original data, including tissue-specific information. While the latent space captures the low-dimensional representation of the input data, it may not directly correspond to specific classes or tissue types in the case of medical imaging data. By training the VAE to accurately reconstruct the input, the decoder learns to extract relevant features for tissue classification, in addition, combined with appropriate loss functions and training procedures, it helps in learning a latent space representation that exhibits better class separability. The application of the classification derived from the latent space for the lesions yielded acceptable local tissue scores that agreed well with the original visual assessment of areas of free lumen and the presence of small stenosis (lesion sample #1), predominantly or partially patent lumen (lesion samples #2, #3 and #5) and complex lesion composition (lesions #4). For the latter, it is of note in this feasibility study that the tissue score identified by the AI-algorithm of a region mostly occluded by hard tissue was sandwiched between two regions mostly occluded by soft tissue potentially reflecting the development of PAD lesions from simple (lesion #1) to complex (lesion #4). Beyond the established feasibility as described above, further evaluation of this algorithm is necessary when expanding to additional lesions and tissue types. UTE imaging has been shown to be sensitive for differentiating calcium from other tissues with short T2* values, which do not show intensity in conventional T1w or T2w images. While further validation of the color attribution to tissues as presented here may be warranted, our feasibility study demonstrated that tissues with signal from UTE and not from traditional T1w and T2w contrasts can be uniquely identified due to the separation of images in latent space of the VAE algorithm and that in particular the AI-algorithm VAE is suitable to separate hard and soft tissue types and their location in a PAD lesion imaged with a dedicated high-resolution MRI-histology protocol.

### 4.1. Limitations

The limitations of this study include small number of lesions. While providing sufficient training data for the VAE, further expansion with more lesions and images may provide a more distinct distribution of the data in latent space. Retraining the VAE with more lesions may improve the quality of the reconstructed images as well as the classification. Expanding the color space in the RGB images may lead to refinement of the classification, potentially including the free lumen as an automated fast method for planning the intervention. Also, we used a 2D latent space in our custom-made VAE. Using a higher-dimensional latent space may be more feasible and beneficial in non-linear manifolds. Finally, this method is a “semi-objective” measure of assessing feasibility. In the future, incorporating additional objective factors could further contribute to the consolidation of our findings.

### 4.2. Novelties

(1) We were able to extract a sufficient number of images for training a 2D CNN VAE algorithm from multi-contrast high-resolution MRI images of PAD lesions.
(2) In our custom-made AI algorithm, we adapted the structure of the CNN layers to enable image reconstruction of multi-contrast MRI lesion tissue components.
(3) The multi-contrast MRI lesion images were sorted into separate classes based on the presence or absence of hard tissue types, with corresponding tissue scores assigned.
(4) The tissue scores were evaluated by visually assessing the pseudo-color multi-contrast MRI coronal images in a concurrent display.

## 5. Conclusions

In our feasibility study, we demonstrated a novel application of the Variational Autoencoder algorithm combined with 2D convolutional networks for analyzing multi-contrast MRI images. Our custom implementation successfully sorted relevant image features, specifically the presence or absence of hard tissue types, within the latent space. By partitioning the latent space accordingly, we were able to identify the presence of hard tissues in PAD lesions.

For future endeavors, it is highly recommended and promising to expand the algorithm further and validate its performance through supervised classification techniques. This would enhance the algorithm’s capabilities and provide a more rigorous evaluation of its effectiveness.

## Figures and Tables

**Table 1:** Relative percentages of the tissue classes for each lesion sample (#1–#5).

Sample Number	Tissue Class I (%)	Tissue Class II (%)	Tissue Class III (%)	Tissue Class IV (%)
#1	100	0	0	0
#2	4.2	75.9	19.9	0
#3	9.8	72.2	18	0
#4	0.2	46.3	33.5	20
#5	5.8	69.6	24.7	0

## Data Availability

The data presented in this study are available on request from the corresponding author.

## References

[1] Bradbury A.W., Adam D.J., Bell J., Forbes J.F., Fowkes F.G.R., Gillespie I., Ruckley C.V., Raab G.M. (2010). Bypass versus Angioplasty in Severe Ischaemia of the Leg (BASIL) trial: Analysis of amputation free and overall survival by treatment received. J. Vasc. Surg..

[2] Farber A., Menard M.T., Conte M.S., Kaufman J.A., Powell R.J., Choudhry N.K., Hamza T.H., Assmann S.F., Creager M.A., Cziraky M.J. (2022). Surgery or Endovascular Therapy for Chronic Limb-Threatening Ischemia. N. Engl. J. Med..

[3] Roy T.L., Chen H.-J., Dueck A.D., Wright G.A. (2018). Magnetic resonance imaging characteristics of lesions relate to the difficulty of peripheral arterial endovascular procedures. J. Vasc. Surg..

[4] Conte M.S., Bradbury A.W., Kolh P., White J.V., Dick F., Fitridge R., Mills J.I., Ricco J.-B., Suresh K.R., Murad M.H. (2019). Global vascular guidelines on the management of chronic limb-threatening ischemia. J. Vasc. Surg..

[5] Sharma S., Boujraf S., Bornstedt A., Hombach V., Ignatius A., Oberhuber A., Rasche V. (2010). Quantification of Calcifications in Endarterectomy Samples by Means of High-Resolution Ultra-Short Echo Time Imaging. Investig. Radiol..

[6] Takahashi M., Takehara Y., Fujisaki K., Okuaki T., Fukuma Y., Tooyama N., Ichijo K., Amano T., Goshima S., Naganawa S. (2021). Three Dimensional Ultra-short Echo Time MRI Can Depict Cholesterol Components of Gallstones Bright. Magn. Reson. Med. Sci..

[7] Yassin A., Pedrosa I., Kearney M., Genega E., Rofsky N.M., Lenkinski R.E. (2012). In Vitro MR Imaging of Renal Stones with an Ultra-short Echo Time Magnetic Resonance Imaging Sequence. Acad. Radiol..

[8] Finkenstaedt T., Biswas R., Abeydeera N.A., Siriwanarangsun P., Healey R., Statum S., Bae W.C., Chung C.B. (2019). Ultrashort Time to Echo Magnetic Resonance Evaluation of Calcium Pyrophosphate Crystal Deposition in Human Menisci. Investig. Radiol..

[9] Dou W., Mastrogiacomo S., Veltien A., Alghamdi H.S., Walboomers X.F., Heerschap A. (2018). Visualization of calcium phosphate cement in teeth by zero echo time ^1^H MRI at high field. NMR Biomed..

[10] Siu A.G., Ramadeen A., Hu X., Morikawa L., Zhang L., Lau J.Y.C., Liu G., Pop M., Connelly K.A., Dorian P. (2015). Characterization of the ultrashort-TE (UTE) MR collagen signal. NMR Biomed..

[11] Roy T., Liu G., Shaikh N., Dueck A.D., Wright G.A. (2017). Puncturing Plaques. J. Endovasc. Ther. Off. J. Int. Soc. Endovasc. Spec..

[12] Gore J.C. (2020). Artificial intelligence in medical imaging. Magn. Reson. Imaging.

[13] Zhou L.Q., Wang J.Y., Yu S.Y., Wu G.G., Wei Q., Deng Y.B., Wu X.L., Cui X.W., Dietrich C.F. (2019). Artificial intelligence in medical imaging of the liver. World J. Gastroenterol..

[14] Chassagnon G., Vakalopoulou M., Paragios N., Revel M.-P. (2020). Artificial intelligence applications for thoracic imaging. Eur. J. Radiol..

[15] Le E., Wang Y., Huang Y., Hickman S., Gilbert F. (2019). Artificial intelligence in breast imaging. Clin. Radiol..

[16] Currie G., Hawk K.E., Rohren E., Vial A., Klein R. (2019). Machine Learning and Deep Learning in Medical Imaging: Intelligent Imaging. J. Med. Imaging Radiat. Sci..

[17] Nensa F., Demircioglu A., Rischpler C. (2019). Artificial Intelligence in Nuclear Medicine. J. Nucl. Med..

[18] Wagner J.B. (2019). Artificial Intelligence in Medical Imaging. Radiol. Technol..

[19] Seah J., Brady Z., Ewert K., Law M. (2021). Artificial intelligence in medical imaging: Implications for patient radiation safety. Br. J. Radiol..

[20] Shen Y.-T., Chen L., Yue W.-W., Xu H.-X. (2021). Artificial intelligence in ultrasound. Eur. J. Radiol..

[21] Chen X., Huo X., Wu Z., Lu J. (2021). Advances of Artificial Intelligence Application in Medical Imaging of Ovarian Cancers. Chin. Med. Sci. J..

[22] Streiner D.L., Saboury B., Zukotynski K.A. (2022). Evidence-Based Artificial Intelligence in Medical Imaging. PET Clin..

[23] Singh A., Ogunfunmi T. (2021). An Overview of Variational Autoencoders for Source Separation, Finance, and Bio-Signal Applications. Entropy.

[24] Marino J. (2021). Predictive Coding, Variational Autoencoders, and Biological Connections. Neural Comput..

[25] Ye F., Bors A.G. (2021). Lifelong Mixture of Variational Autoencoders. IEEE Trans. Neural Netw. Learn. Syst..

[26] Gomari D.P., Schweickart A., Cerchietti L., Paietta E., Fernandez H., Al-Amin H., Suhre K., Krumsiek J. (2022). Variational autoencoders learn transferrable representations of metabolomics data. Commun. Biol..

[27] Barrejon D., Olmos P.M., Artes-Rodriguez A. (2022). Medical Data Wrangling with Sequential Variational Autoencoders. IEEE J. Biomed. Health Informatics.

[28] Perl Y.S., Bocaccio H., Pérez-Ipiña I., Zamberlán F., Piccinini J., Laufs H., Kringelbach M., Deco G., Tagliazucchi E. (2020). Generative Embeddings of Brain Collective Dynamics Using Variational Autoencoders. Phys. Rev. Lett..

[29] Baucum M., Khojandi A., Vasudevan R. (2021). Improving Deep Reinforcement Learning with Transitional Variational Autoencoders: A Healthcare Application. IEEE J. Biomed. Health Inform..

[30] Uzunova H., Schultz S., Handels H., Ehrhardt J. (2019). Unsupervised pathology detection in medical images using conditional variational autoencoders. Int. J. Comput. Assist. Radiol. Surg..

[31] Edupuganti V., Mardani M., Vasanawala S., Pauly J. (2021). Uncertainty Quantification in Deep MRI Reconstruction. IEEE Trans. Med. Imaging.

[32] Everett R., Flores K.B., Henscheid N., Lagergren J., Larripa K., Li D., Nardini J.T., Nguyen P.T.T., Pitman E.B., Rutter E.M. (2020). A tutorial review of mathematical techniques for quantifying tumor heterogeneity. Math. Biosci. Eng..

[33] Ahmad B., Sun J., You Q., Palade V., Mao Z. (2022). Brain Tumor Classification Using a Combination of Variational Autoencoders and Generative Adversarial Networks. Biomedicines.

[34] Tschuchnig M.E., Zillner D., Romanelli P., Hercher D., Heimel P., Oostingh G.J., Couillard-Després S., Gadermayr M. (2021). Quantification of anomalies in rats’ spinal cords using autoencoders. Comput. Biol. Med..

[35] Baur C., Denner S., Wiestler B., Navab N., Albarqouni S. (2021). Autoencoders for unsupervised anomaly segmentation in brain MR images: A comparative study. Med. Image Anal..

[36] Nakao T., Hanaoka S., Nomura Y., Murata M., Takenaga T., Miki S., Watadani T., Yoshikawa T., Hayashi N., Abe O. (2021). Unsupervised Deep Anomaly Detection in Chest Radiographs. J. Digit. Imaging.

[37] Pinaya W.H., Tudosiu P.-D., Gray R., Rees G., Nachev P., Ourselin S., Cardoso M.J. (2022). Unsupervised brain imaging 3D anomaly detection and segmentation with transformers. Med. Image Anal..

[38] Geenjaar E., Lewis N., Fu Z., Venkatdas R., Plis S., Calhoun V. (2021). Fusing multimodal neuroimaging data with a variational autoencoder. Annu. Int. Conf. IEEE Eng. Med. Biol. Soc..

[39] Guleria H.V., Luqmani A.M., Kothari H.D., Phukan P., Patil S., Pareek P., Kotecha K., Abraham A., Gabralla L.A. (2023). Enhancing the Breast Histopathology Image Analysis for Cancer Detection Using Variational Autoencoder. Int. J. Environ. Res. Public Health.

[40] Balaji K. (2023). Image Augmentation based on Variational Autoencoder for Breast Tumor Segmentation. Acad. Radiol..

[41] Chatterjee S., Maity S., Bhattacharjee M., Banerjee S., Das A.K., Ding W. (2023). Variational Autoencoder Based Imbalanced COVID-19 Detection Using Chest X-Ray Images. New Gener. Comput..

[42] Zhou Q., Wang S., Zhang X., Zhang Y.-D. (2022). WVALE: Weak variational autoencoder for localisation and enhancement of COVID-19 lung infections. Comput. Methods Programs Biomed..

[43] Chatterjee S., Sciarra A., Dünnwald M., Tummala P., Agrawal S.K., Jauhari A., Kalra A., Oeltze-Jafra S., Speck O., Nürnberger A. (2022). StRegA: Unsupervised anomaly detection in brain MRIs using a compact context-encoding variational autoencoder. Comput. Biol. Med..

[44] Xu W., Tan Y. (2020). Semisupervised Text Classification by Variational Autoencoder. IEEE Trans. Neural. Netw. Learn. Syst..

[45] Chen J., Du L., Liao L. (2022). Discriminative Mixture Variational Autoencoder for Semisupervised Classification. IEEE Trans. Cybern..

[46] Harefa E., Zhou W. (2021). Performing sequential forward selection and variational autoencoder techniques in soil classification based on laser-induced breakdown spectroscopy. Anal. Methods.

[47] Mansour R.F., Escorcia-Gutierrez J., Gamarra M., Gupta D., Castillo O., Kumar S. (2021). Unsupervised Deep Learning based Variational Autoencoder Model for COVID-19 Diagnosis and Classification. Pattern Recognit. Lett..

[48] Yang Y., Zheng K., Wu C., Yang Y. (2019). Improving the Classification Effectiveness of Intrusion Detection by Using Improved Conditional Variational AutoEncoder and Deep Neural Network. Sensors.

[49] Way G.P., Greene C.S. (2018). Extracting a biologically relevant latent space from cancer transcriptomes with variational autoencoders. Pac. Symp. Biocomput..

[50] Fedorov A., Beichel R., Kalpathy-Cramer J., Finet J., Fillion-Robin J.-C., Pujol S., Bauer C., Jennings D., Fennessy F., Sonka M. (2012). 3D Slicer as an image computing platform for the Quantitative Imaging Network. Magn. Reson. Imaging.

[51] Schneider C.A., Rasband W.S., Eliceiri K.W. (2012). NIH Image to ImageJ: 25 Years of image analysis. Nat. Methods.

[52] Rocha-Singh K.J., Zeller T., Jaff M.R. (2014). Peripheral arterial calcification: Prevalence, mechanism, detection, and clinical implications. Catheter. Cardiovasc. Interv..

[53] Fanelli F., Cannavale A., Gazzetti M., Lucatelli P., Wlderk A., Cirelli C., D’adamo A., Salvatori F.M. (2014). Calcium Burden Assessment and Impact on Drug-Eluting Balloons in Peripheral Arterial Disease. Cardiovasc. Interv. Radiol..

[54] Mohebali J., Patel V.I., Romero J.M., Hannon K.M., Jaff M.R., Cambria R.P., LaMuraglia G.M. (2015). Acoustic shadowing impairs accurate characterization of stenosis in carotid ultrasound examinations. J. Vasc. Surg..

[55] Roy T., Forbes T., Wright G., Dueck A. (2015). Burning Bridges: Mechanisms and Implications of Endovascular Failure in the Treatment of Peripheral Artery Disease. J. Endovasc. Ther..

[56] Roy T.L., Forbes T.L., Dueck A.D., Wright G.A. (2018). MRI for peripheral artery disease: Introductory physics for vascular physicians. Vasc. Med..

[57] Edelman R.R., Flanagan O., Grodzki D., Giri S., Gupta N., Koktzoglou I. (2015). Projection MR imaging of peripheral arterial calcifications: Projection MR Imaging of Peripheral Arterial Calcifications. Magn. Reson. Med..

[58] Karolyi M., Seifarth H., Liew G., Schlett C.L., Maurovich-Horvat P., Dai G., Huang S., Goergen C.J., Hoffmann U., Sosnovik D.E. (2012). Classification of human coronary atherosclerotic plaques with T1, T2 and Ultrashort TE MRI. J. Cardiovasc. Magn. Reson..

[59] Saba L., Sanagala S.S., Gupta S.K., Koppula V.K., Johri A.M., Khanna N.N., Mavrogeni S., Laird J.R., Pareek G., Miner M. (2021). Multimodality carotid plaque tissue characterization and classification in the artificial intelligence paradigm: A narrative review for stroke application. Ann. Transl. Med..

[60] Lanzafame L.R.M., Bucolo G.M., Muscogiuri G., Sironi S., Gaeta M., Ascenti G., Booz C., Vogl T.J., Blandino A., Mazziotti S. (2023). Artificial Intelligence in Cardiovascular CT and MR Imaging. Life.

[61] Azeez M., Laivuori M., Tolva J., Linder N., Lundin J., Albäck A., Venermo M., Mäyränpää M.I., Lokki M.L., Lokki A.I. (2022). High relative amount of nodular calcification in femoral plaques is associated with milder lower extremity arterial disease. BMC Cardiovasc. Disord..

